# Measurable residual disease quantification in adult patients with *KMT2A*-rearranged acute lymphoblastic leukemia

**DOI:** 10.1038/s41375-024-02209-7

**Published:** 2024-03-22

**Authors:** Thomas Burmeister, Aeint-Steffen Ströh, Britta Kehden, Heiko Trautmann, Claus Meyer, Rolf Marschalek, Patrizia Larghero, Stefan Schwartz, Björn Steffen, Bernd Spriewald, Thomas Heinicke, Nadja Jäkel, Jörg Westermann, Kathrin Nachtkamp, Andreas Viardot, Max S. Topp, Martin Neumann, Claudia D. Baldus, Nicola Gökbuget, Monika Brüggemann

**Affiliations:** 1 Department of Hematology, Oncology and Tumor Immunology, CVK, Charité—Universitätsmedizin, corporate member of Freie Universität Berlin and Humboldt-Universität zu Berlin, Berlin, Germany; 2 University Cancer Center Schleswig-Holstein (UCCSH), University Hospital Schleswig-Holstein, Kiel, Germany; 3 Department of Medicine II, Hematology and Oncology, University Hospital Schleswig-Holstein, Kiel, Germany; 4 Diagnostic Center of Acute Leukemia (DCAL), Institute of Pharmaceutical Biology, Goethe University, Frankfurt, Germany; 5 Department of Hematology, Oncology and Tumor Immunology, CBF, Charité Universitätsmedizin, corporate member of Freie Universität Berlin and Humboldt-Universität zu Berlin, Berlin, Germany; 6 Department of Medicine II, Goethe University, University Hospital, Frankfurt, Germany; 7 University Hospital Erlangen, Department of Internal Medicine 5, Hematology and Oncology, Erlangen, Germany; 8 Department of Hematology and Oncology, Otto-von-Guericke University Hospital, Magdeburg, Germany; 9 Department of Hematology, Oncology, University Hospital, Halle/Saale, Germany; 10 Department of Hematology, Oncology, University Hospital of Düsseldorf, Düsseldorf, Germany; 11 Department of Hematology, Oncology, University Hospital Ulm, Ulm, Germany; 12 Department of Hematology, Oncology, University Hospital Würzburg, Würzburg, Germany

**Keywords:** Acute lymphocytic leukaemia, Risk factors, Disease-free survival, Cancer genetics

## To the Editor:

In adult ALL, 5–10% of patients show *KMT2A* translocations (*KMT2A* rearrangements) with only a few secondary alterations, implicating it as a leukemia-initiating factor [1, 2]. Approximately 95% of all fusions in adult ALL are *KMT2A*::*AFF1* or *KMT2A*::*MLLT1* [3]. *KMT2A*-rearranged adult ALL patients are generally considered high-risk and are treated with intensified therapy, including allogeneic hematopoietic stem cell transplantation (SCT) [4]. Current ALL treatment protocols are often guided by measurable residual disease (MRD)-based risk stratification [4–8], however, limited data are available regarding the prognostic value of MRD in adult ALL with *KMT2A* rearrangement. In infant *KMT2A*-rearranged ALL, more reliable MRD data were obtained using the individual *KMT2A* breakpoints as molecular MRD target as compared to *IG*/*TR* [6, 9–11], but no such comparisons have been made in adult ALL. We evaluated the impact of MRD on disease-free survival (DFS) and overall survival (OS) in a cohort of 156 *KMT2A*-rearranged adult patients and compared *IG*/*TR*- and *KMT2A*-based MRD levels in 46 patients.

In total, 769 bone marrow and/or peripheral blood samples from 193 adult ALL patients with *KMT2A* rearrangement (175 *KMT2A*::*AFF1*, 13 *KMT2A*::*MLLT1*, 1 *KMT2A*::*MLLT3*, 4 *KMT2A+* unspecified) obtained between 2001 and 2021 were available for longitudinal MRD measurements. All patients were treated according to different protocols of the German Multicenter ALL (GMALL) study group and gave their informed consent to further scientific investigations on residual material. Patients with *KMT2A*::*AFF1* aged up to 55 years were assigned to the high-risk group and were candidates for SCT in first CR after consolidation I. Immunophenotyping and MRD measurement with real-time PCR based on *KMT2A* fusion genes and clonal *IG*/*TR* gene rearrangements were performed in central laboratories as previously described [10, 11]. MRD measurements were interpreted according to EuroMRD guidelines [12]. MRD results were considered discordant if positivity/negativity discordance in the same sample was evidenced. For the evaluation of DFS and OS, MRD levels were compared at three different time points: end of induction I, after induction II/ pre-consolidation I, post-consolidation I/pre SCT (around week 16) (Fig. S2) [11]. MRD levels were classified as *molecular response* (MRD < 10^–4^ or negative), *molecular failure with low MRD* (≥10^–4^ and <10^–2^), and *high MRD* (≥10^–2^). Further statistical details are provided in the online supplement to this letter.

## *KMT2A*-based versus *IG/TR*-based MRD

**Fig. 1:**
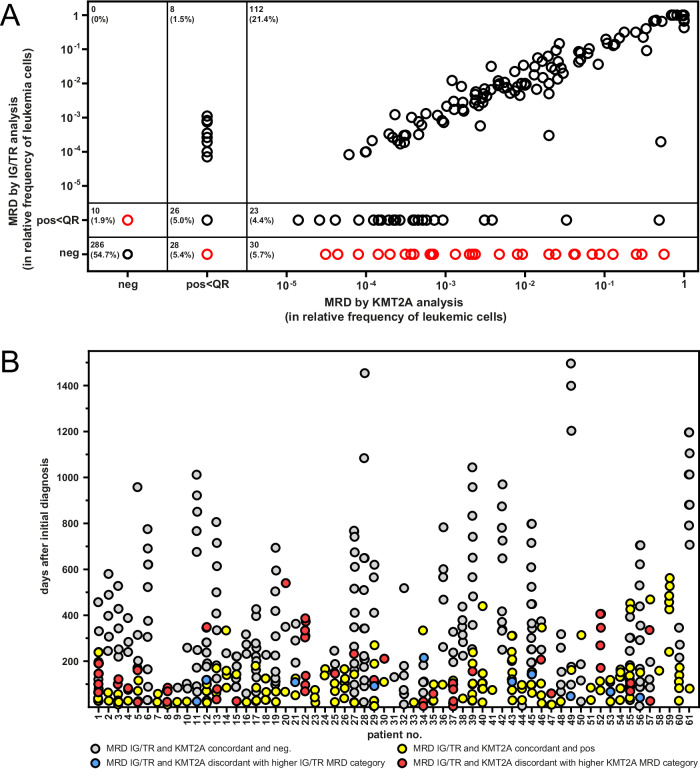
Comparison of *KMT2A*-based and *IG*/*TR*-based MRD measurements. **A** Comparison of MRD levels with *KMT2A* and *IG*/*TR* targets in *KMT2A*-rearranged adult ALL patients. MRD measurements with data on both *KMT2A* and *IG*/*TR* were available from 46 patients totaling 373 sample pairs from peripheral blood or bone marrow aspirates. MRD levels were plotted against each other from negative (neg), positive (pos)< quantifiable range (QR), and quantifiable range in logarithmic format. Black circles represent MRD concordant samples and red circles discordant samples. **B** Comparison of *KMT2A* and *IG*/*TR* MRD levels over time. All MRD-levels (*n* = 523) with data on both *IG/TR* and *KMT2A* were sorted into four groups (gray color *IG*/*TR* and *KMT2A* MRD level concordant and negative (neg.), blue color *IG*/*TR* and *KMT2A* MRD level discordant with higher *IG*/*TR* MRD category, yellow color *IG*/*TR* and *KMT2A* MRD level concordant and positive, and red color *IG*/*TR* and *KMT2A* MRD level discordant with higher *KMT2A* MRD category) and plotted against days after initial diagnosis (ID).

We analyzed 193 patients with *KMT2A*-rearranged ALL with median age at diagnosis of 42.5 years (18.0-76.8), and 63.0% being females. All 187 immunophenotypically characterized patients showed a CD10-negative B cell precursor ALL (146 cyIg^neg^, 41 cyIg^pos^). Parallel MRD data of both, *KMT2A* and *IG*/*TR*, were available for 46 patients, totaling 274 MRD data pairs from bone marrow and 99 from peripheral blood. Both methods show good agreement (Table S2; Fig. S1). 197/373 (52.8%) samples were MRD-negative with both methods, 84/373 (22.5%) were congruently positive within quantifiable range (QR), and 22/373 (5.9%) were positive below QR in both MRD targets (Fig. 1A). 18/373 (4.8%) were quantifiable MRD-positive only using *KMT2A*, whereas *IG/TR* MRD showed positivity below QR of the method, in 6/373 cases (1.6%) it was the other way around. The remaining 46/373 (13.0%) samples were classified as discordant, with 38/373 (10.2%) being *KMT2A*-rearranged and *IG/TR*^*neg*^, with 24/46 samples showing quantifiable *KMT2A* MRD positivity. Only 8/373 (2.1%) were *KMT2A*^*ne*g^ and *IG/TR*^*pos*^ (*P* < 0.0001), none of them showing quantifiable *IG/TR* MRD positivity (Table S1). Discordant samples with the higher *KMT2A* MRD were detected at least once in 15/46 (32.6%) patients during therapy and follow-up, whereas a higher *IG*/*TR* MRD was detected in 8/46 (17.4%) patients (Fig. 1B). These results are consistent with other studies on *KMT2A*-rearranged ALL, where *IG*/*TR* rearrangements at diagnosis were often oligo- or subclonal and underly clonal evolution [9]. Usage of subclonal IG/TR markers or a loss of the MRD marker due to RAG-mediated clonal evolution may lead to false negative results or underestimation of MRD values. In contrast, the *KMT2A* break cannot get lost because it is an early event and a leukemia-defining molecular hallmark.

## Prognostic significance of MRD

**Fig. 2:**
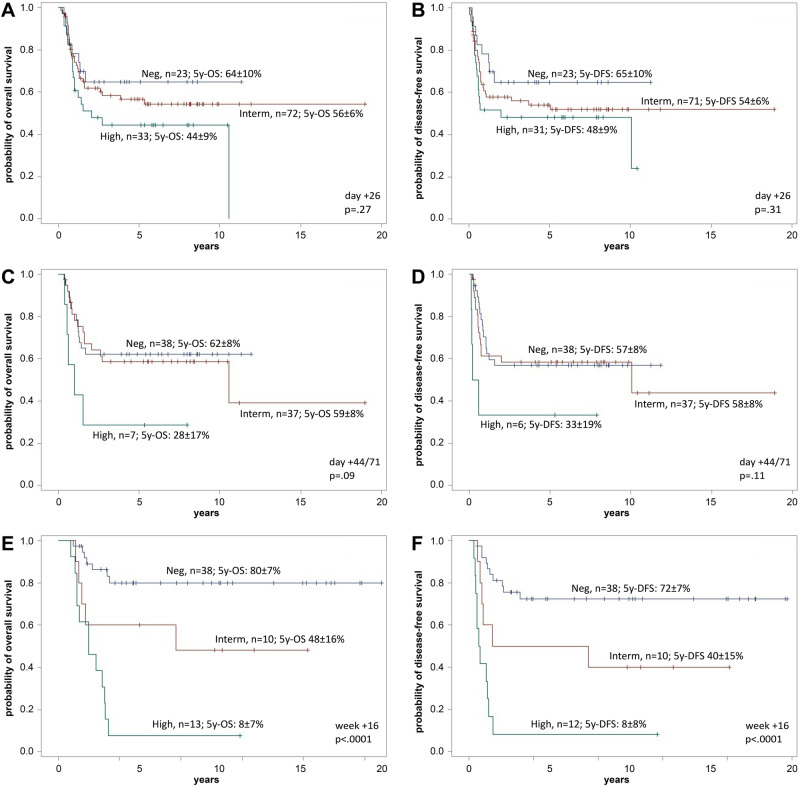
Overall survival and disease-free survival of patients at different time points. Prognostic impact of measurable residual disease (MRD) levels at the end of induction I (**A** + **B**), pre-consolidation I (**C** + **D**), and post-consolidation I (**E** + **F**). Data shown by Kaplan–Meier estimates of overall survival (**A**, **C**, **E**) and disease-free survival (**B**, **D**, **F**). MRD results were classified as low (blue color; neg; <10^–4^), intermediate (red color; interm; <10^–2^), and high MRD levels (green color; ≥10^–2^). Information on DFS was not available for some patients (day+26: 3 pts., day+44/71: 1 pt., w +16: 1 pt.).

Data for DFS and OS in remission were available for 156/193 patients, with MRD being assessed using *IG*/*TR* and/or *KMT2A* at the end of induction I (*n* = 140), after induction II/ pre-consolidation I (*n* = 149) and after consolidation I (*n* = 68). After induction I, MRD levels did not predict outcome with 5-year DFS and OS (*P* = 0.31 and *P* = 0.27) (Fig. 2A+B). At pre-consolidation I, MRD levels did not predict outcome with 5-year DFS but patients with high MRD (≥10^–2^) levels had a significantly poorer OS. 5-year OS was 62% (95% CI: 54 to 70), 59% (95% CI: 52 to 67), and 28% (95% CI: 11 to 45) for patients with molecular response, and molecular failure with low or high MRD (*P* = 0.09) (Fig. 2C+D). After consolidation I significant differences were found in both DFS and OS, and MRD levels predicted outcome with 5-year DFS of 72% (95% CI, 64 to 80), 40% (95% CI, 25 to 55), and 8% (95% CI, 0 to 16) and 5-year OS of 80% (95% CI, 73 to 88), 48% (95% CI, 32 to 64), and 8% (95% CI, 1 to 15) for patients with molecular response, and molecular failure with low (MRD ≥ 10^–4^ and <10^–2^) and high (≥10^–2^) MRD levels (*P* < 0.0001) (Fig. 2E+F). These findings demonstrate that high MRD levels at post-consolidation I in adult *KMT2A*-rearranged ALL are clearly unfavorable in terms of OS and DFS prior to SCT. Strikingly, early MRD after induction I was not predictive for treatment outcome, which contrasts with published data from other ALL molecular subgroups where early MRD has shown clear prognostic significance [5, 7, 11]. It is possible that this observation reflects the same mechanism that has been described for infant *KMT2A*-rearranged ALL: In a study by Stutterheim et al. [13], MRD after induction was prognostically relevant only if followed by a lymphoid-style consolidation but not with a myeloid-style type consolidation. In our patient cohort allogeneic SCT was performed in the majority of patients (72%) which may abolish the prognostic effect of postinduction MRD response. However, patients with molecular failure prior to SCT still had poorer outcome. This supports the GMALL approach to offer a targeted therapy with blinatumomab to all patients with molecular failure after consolidation I to eradicate MRD before SCT [14]. However, patients with *KMT2A*-rearranged ALL occasionally show CD19 antigen loss after blinatumomab and blinatumomab may also be less effective than in non-*KMT2A*-rearranged ALL, due to lower CD19 expression.

## Myeloid coexpression and MRD response

In the Interfant-06 study, patients with myeloid coexpression had significantly higher MRD levels at the end of induction and benefitted from subsequent myeloid-style consolidation [13]. In our cohort data were available in 96 patients for both, detailed immunophenotype and MRD. Expression of at least one myeloid marker (CD13, CD15, CD65s, CD33) was detected in 77 (80.2%) patients. We observed no significant differences in MRD response at end of induction I, pre- or post-consolidation in patients with or without myeloid co-expression (Fig. S3; Table S3).

In conclusion, our study suggests that in adult *KMT2A*-rearranged ALL the *KMT2A* genomic fusion breakpoint has clear technical advantages as MRD target, as has also recently been reported by Kim et al. [15]. However, patient numbers in our study were too small to prove a clinical impact of MRD discordance between these two methods. The MRD status has a very strong prognostic value in DFS and OS post-consolidation I in a transplant-oriented regimen. The optimal therapy of patients with treatment failure or MRD persistence is under investigation. Particularly the term “myeloid-style therapy” needs to be defined more precisely, since most relevant compounds are also part of ALL therapy. More promise probably lies in the use of immunotherapies directed to lymphoid surface markers like CD19.

### Supplementary information

Supplemental material
