# Supplementary material for: Measurable residual disease quantification in adult patients with KMT2A-rearranged acute lymphoblastic leukemia
Source: Leukemia. 2024 Mar 22;38(7):1600–3. doi: 10.1038/s41375-024-02209-7 (PMC11216977; doi:10.1038/s41375-024-02209-7)

Supplementary Appendix

Table of contents

Section		Page
	Supplementary Methods	1
Table S1	Accuracy of *KMT2A* MRD compared with *IG/TR* MRD	1
Table S2	Agreement between *KMT2A* MRD and *IG/TR* MRD	1
Table S3	Myeloid coexpression and MRD response	2
Figure S1	Comparison of MRD levels with *KMT2A* and *IG/TR* targets in *KMT2A*-rearranged adult ALL patients	3
Figure S2	Simplified treatment schema of GMALL therapy recommendations for the high-risk group of adult *KMT2A*-rearranged ALL patients	4
Figure S3	Comparison of MRD levels in context of expression of myeloid markers at the end of induction I, pre-consolidation I, and post-consolidation I	4
Figure S4	Comparison of MRD levels with *KMT2A* and *IG/TR* targets in *KMT2A*-rearranged adult ALL patients from peripheral blood and bone marrow aspirates	5

Supplementary Methods

All MRD measurements utilized in *KMT2A*-based versus *IG/TR*-based MRD comparison had a sensitivity of at least 10^{-4}. The results of MRD assessments with real-time PCR based on *KMT2A* fusion genes and *IG/TR* genes are categorized as positive (positive within quantifiable range and positive not quantifiable), or negative. The accuracy of *KMT2A* RQ-PCR is compared with *IG/TR* RQ-PCR using McNemar's test. In analyzing the agreement between *KMT2A* MRD measurements with *IG/TR* MRD measurements was investigated using Bland-Altman plots and evaluated by Cohen's kappa (κ) for dichotomized results (positive/negative). Interpreting kappa values, we use the guidelines according to Fleiss et al. (2003). Analysis of the influence of myeloid coexpression on MRD was performed using a Kruskal-Wallis test for MRD values and Chi-squared test for MRD categories (low/intermediate/high). All tests were two-sided. In case of multiple established markers of *IG/TR* (and/or *KMT2A* for evaluation of DFS and OS), we used the highest MRD value. We used Kaplan- Meier estimates with two-sided 95% confidence intervals (CI) to describe DFS and OS. Subgroups were compared using log-rank tests. DFS was defined as the time interval between baseline MRD detection to hematologic relapse or death from any cause; patients who lived without relapse were censored at their last follow-up. OS was defined as the time interval between baseline MRD detection to death; patients without recorded death were censored at their last follow-up. McNemar's test, Cohen's kappa (κ), Chi-squared test, and Kruskal-Wallis test were performed using the SPSS software version 26.0.0 (SPSS, Chicago, IL). All other analyses were performed using Statistical Analysis Software (release 9.4) procedures and macros (SAS/STAT User's Guide 14.3; Cary, NC) in the GMALL Study Center

Supplementary Table 1

Accuracy of *KMT2A* MRD compared with *IG/TR* MRD. McNemar's Chi-squared test was used to compare the dichotomous results between *KMT2A* and *IG/TR* MRD. Less samples were diagnosed as positive by *IG/TR* (37.0%) than by *KMT2A* (45.0%), p<0.0001.

	***IG/TR*^{neg}**	***IG/TR*^{pos}**	**Total**	**P value**
***KMT2A*^{neg}**	197 (52.8%)	8 (2.5%)	205 (55.0%)	
***KMT2A*^{pos}**	38 (10.3%)	130 (34.9%)	168 (45.0%)	
**Total**	235 (63.0%)	138 (37.0%)	373 (100%)	< .0001

Supplementary Table 2

Agreement between *KMT2A* MRD and *IG/TR* MRD. Cohen's kappa was computed to assess the agreement between tests MRD positives and negatives (two categories). The strength of agreement between *KMT2A* and *IG/TR* RQ-PCR was classified as good, κ = .729 (95% CI, .670 to .788), p < .0001.

Test results in 2 groups (positive, negative)

	**Cohen's Kappa**	**95% CI**	**P value**
KMT2A vs. *IG/TR* RQ-PCR	.747	0.68 - 0.81	< 0.001

Supplementary Table 3

Myeloid coexpression and MRD response. In our cohort data were available in 96 patients for both, detailed immunophenotype and MRD (at day +26, day +44/71 and/or week +16). Expression of at least one myeloid marker (CD13, CD15, CD65s, CD33) was detected in 77 (80.2%) patients. A chi-square test was used to compare myeloid antigen expression positive (myAg+) and myeloid antigen expression negative (myAg-). Results at timepoint day +26 show no significant between myAg+ and myAg-, $\chi^2(2) = 4.37$, $p = .106$, $V = 0.112$. Results at timepoint day +44/71 show no significant between myAg+ and myAg-, $\chi^2(2) = 1.95$, $p = .521$, $V = 0.378$. Results at timepoint day +44/71 show no significant between myAg+ and myAg-, $\chi^2(2) = 1.94$, $p = .401$, $V = 0.380$. We use Fisher's exact test with at least one expected cell count below five.

day +26

MRD	myAG +	myAG -	Total	P value
molecular response **($<10^{-4}$)**	15	0	15	
molecular failure with low MRD **($\geq 10^{-4}$ and $<10^{-2}$)**	35	12	47	
molecular failure with high MRD **($\geq 10^{-2}$)**	16	6	22	
**Total**	66	18	84	0.106

MRD not available at this timepoint: 12

day +44/71

MRD	myAG +	myAG -	Total	P value
molecular response **($<10^{-4}$)**	11	1	12	
molecular failure with low MRD **($\geq 10^{-4}$ and $<10^{-2}$)**	22	4	26	
molecular failure with high MRD **($\geq 10^{-2}$)**	41	13	54	
**Total**	74	18	92	0.521

MRD not available at this timepoint: 4

week +16

MRD	myAg+	myAg-	Total	P value
molecular response **($<10^{-4}$)**	14	2	16	
molecular failure with low MRD **($\geq 10^{-4}$ and $<10^{-2}$)**	4	0	4	
molecular failure with high MRD **($\geq 10^{-2}$)**	8	3	11	
**Total**	26	5	31	0.401

MRD not available at this timepoint: 65

Supplementary Figure 1

Comparison of MRD levels with *KMT2A* and *IG/TR* targets in *KMT2A*-rearranged adult ALL patients. MRD comparison by Bland-Altman (n=373 with both *IG/TR* and *KTM2A* measurements quantifiable). The dotted line indicates the average difference of 0.15, the dashed lines indicate the Limits of Agreement: -1.44 and 1.45, while the continuous line represents zero difference. To visualize in this plot MRD negative and MRD positive (pos) < quantifiable range (QR), we used MRD highest sensitivity threshold for MRD negative and MRD highest specificity threshold for MRD pos<QR. *IG* = immunoglobulin; *TR* = T-cell receptor.

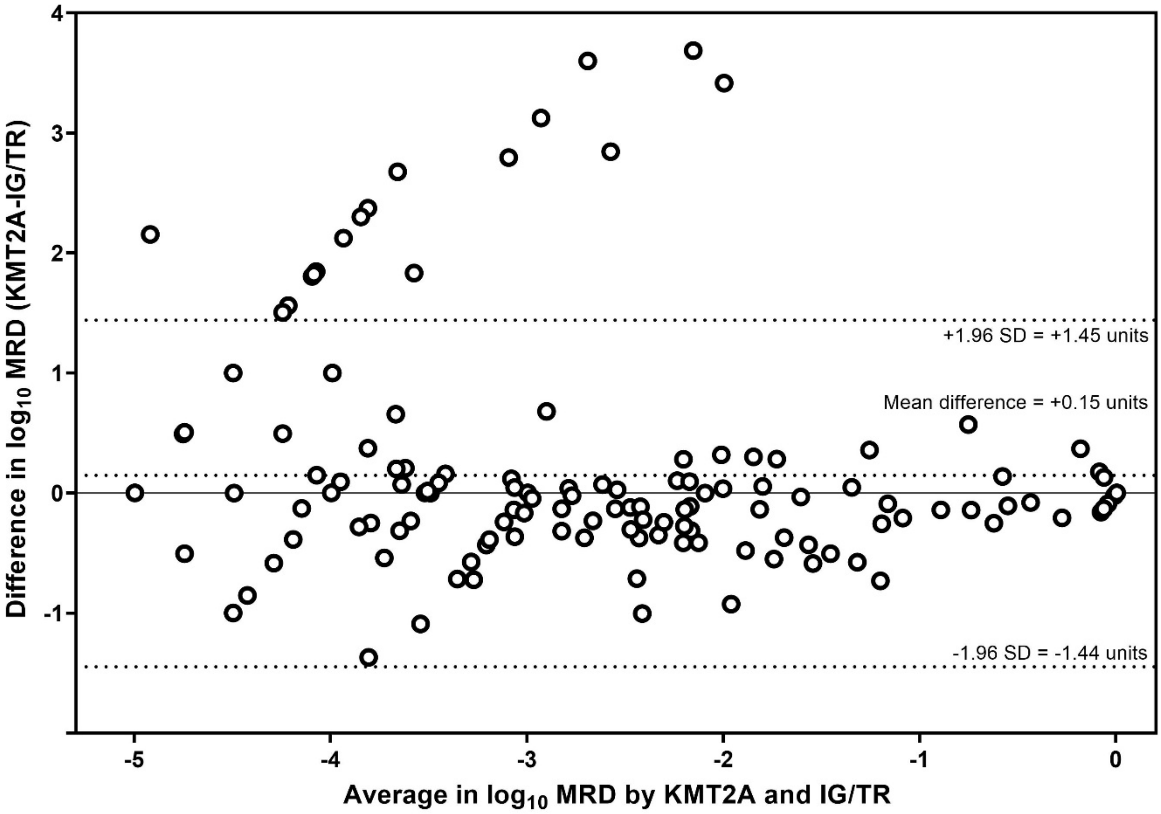

Supplementary Figure 2

Simplified treatment schema of GMALL therapy recommendations for the high-risk group of adult *KMT2A*-rearranged ALL patients. *KMT2A-* rearranged adult patients with t(4;11)/*KMT2A::AFF1-* positive ALL or other *KMT2A* translocations were stratified as high risk. In addition to induction as well as consolidation therapy, the MRD time points used in this study were plotted. MRD = measurable residual disease; ID = initial diagnosis; EOI = end of induction; Pre-C = Pre-Consolidation; Post-C = Post-Consolidation. SCT = stem cell transplantation; d = day; w = week.

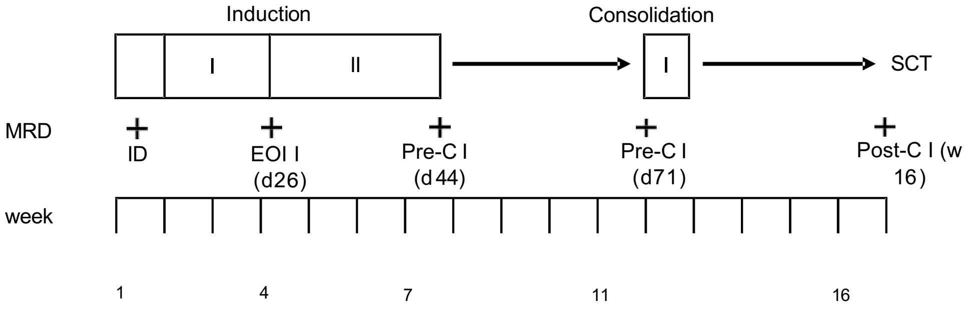

Supplementary Figure 3

Comparison of MRD levels in context of expression of myeloid markers at the end of induction I (A), pre-consolidation I (B), and post-consolidation I (C). 96 patients are available with data for both MRD (at day +26, day +44/71 and/or week +16) and assessment of myeloid immunophenotype. 19 (19.8%) had no expression of a myeloid marker (my-), 77 (80.2%) had expression of at least one myeloid marker (my+). For the time point end of induction I 84, for pre-consolidation I 92, and for post-consolidation I 31 MRD levels with assessment of myeloid immunophenotype were available for data analysis. MRD levels were plotted against myeloid immunphenotype in logarithmic format. In patients with low MRD at the time of end of induction I, 100% showed expression of one or more myeloid markers in the diagnostic sample (n=15/15) versus 74% in patients with intermediate MRD (n=35/47) and 72% in patients with high MRD (n=16/22) (p=0.085) (A). At the time of pre-consolidation I, 92% showed expression of one or more myeloid markers (n=11/12) versus 85% in patients with intermediate MRD (n=22/26) and 76% in patients with high MRD (n=41/54) (p=0.389) (B). At post-consolidation I 87% showed expression of one or more myeloid marker (n=13/15) versus 100% in patients with intermediate MRD (n=4/4) and 75% in patients with high MRD (n=9/12) (p=0.613) (C).

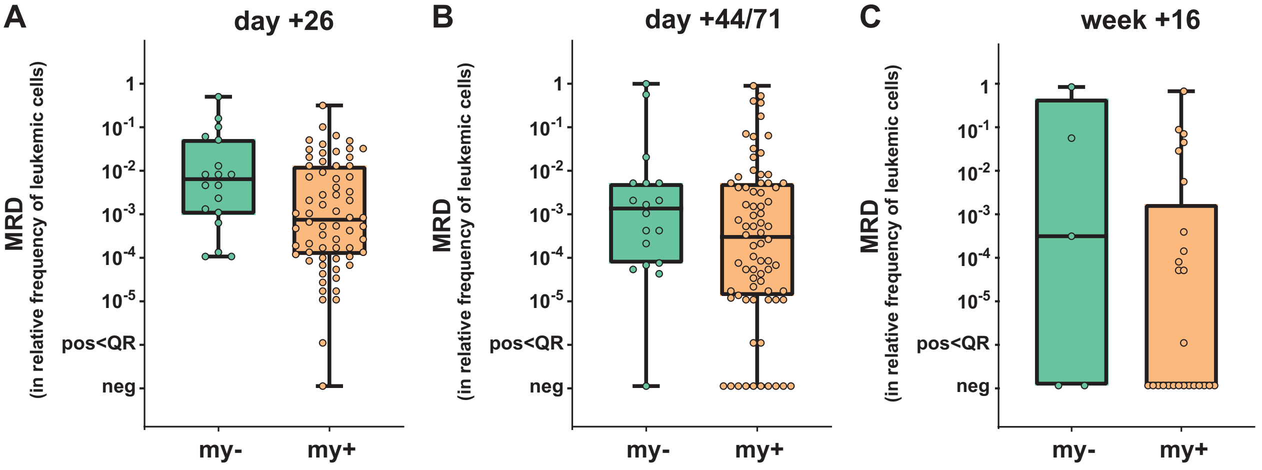

Supplementary Figure 4

Comparison of MRD levels with *KMT2A* and *IG*/*TR* targets in *KMT2A*-rearranged adult ALL patients from peripheral blood (A) and bone marrow aspirates (B). MRD measurements with data on both *KMT2A* and *IG*/*TR* were available from 46 patients totaling 373 sample pairs from peripheral blood (A) or bone marrow aspirates (B). MRD levels were plotted against each other from negative (neg), positive (pos)< quantifiable range (QR), and quantifiable range in logarithmic format. Black dots represent MRD results concordant samples and red dots discordant samples.

A

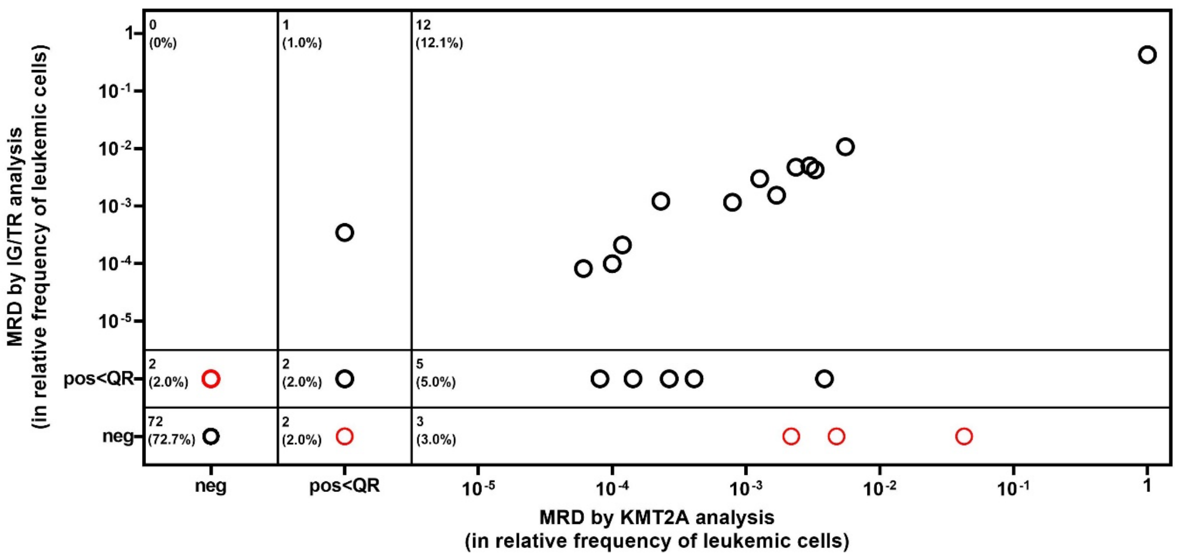

B

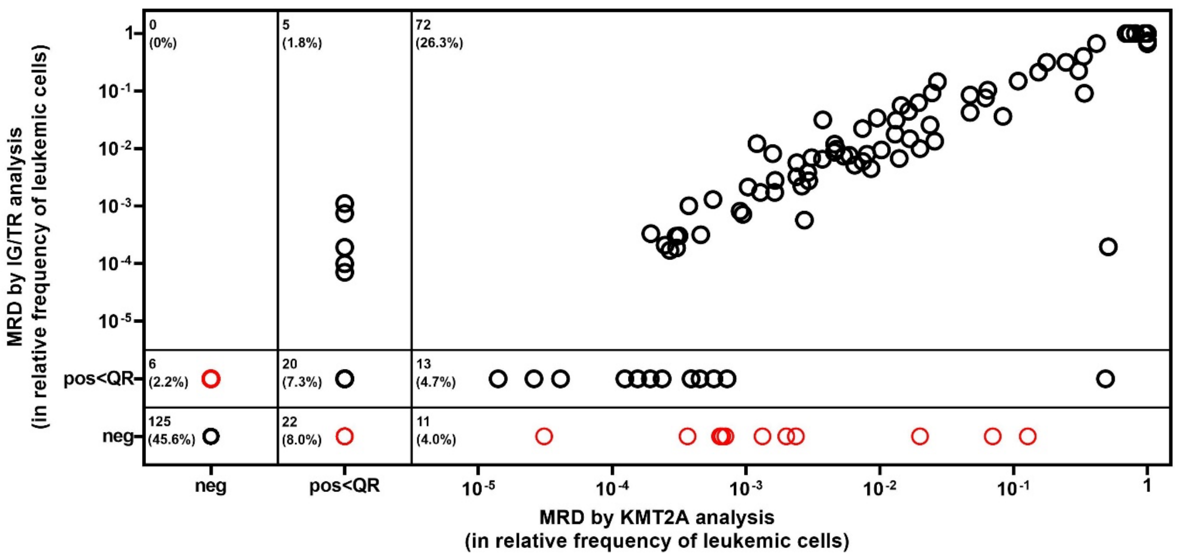

Supplement: Supplementary file 1 — Supplemental material [file 41375_2024_2209_MOESM1_ESM.pdf]
